# Correlation between PD-L1 expression and clinical pathology, immunobiological markers, and prognosis in gastroenteropancreatic neuroendocrine neoplasms: a systematic review and meta-analysis

**DOI:** 10.3389/fimmu.2026.1772011

**Published:** 2026-02-12

**Authors:** Qiming Zheng, Shangbo Jin, Xinyue Xie, Qingjun Guo, Chiyi Chen, Wentao Jiang

**Affiliations:** 1 Department of General Surgery/Transplantation, First Central Hospital of Tianjin Medical University, Tianjin, China; 2 Department of Liver Transplantation, Tianjin First Central Hospital, Tianjin, China; 3 Tianjin Key Laboratory of Molecular Diagnosis and Treatment of Liver Cancer, Tianjin First Center Hospital, Tianjin, China; 4 Tianjin Key Laboratory for Organ Transplantation, Tianjin First Center Hospital, Tianjin, China; 5 Department of Hepatobiliary Pancreatic Spleen Surgery, Baoji People’s Hospital, Baoji, Shaanxi, China; 6 Institute of Transplantation Medicine, Nankai University, Tianjin, China

**Keywords:** gastroenteropancreatic, immunotherapy, neuroendocrine neoplasms, prognosis, programmed cell death ligand 1

## Abstract

**Background:**

Advanced gastroenteropancreatic neuroendocrine neoplasms (GEP-NENs) have limited therapeutic options. The role of programmed death-ligand 1 (PD-L1) in their clinicopathology, immune markers, and prognosis remains controversial. This meta-analysis aimed to systematically clarify these relationships.

**Methods:**

We searched Medline/PubMed, Web of Science, Embase, and Cochrane Library from inception to November 2025 for studies on PD-L1 expression in GEP-NENs. Two researchers independently extracted data and assessed quality via Newcastle-Ottawa Scale (NOS). Pooled analyses were conducted using Stata 17.0, with odds ratio (OR)/hazard ratio (HR) and 95% confidence interval (CI) as effect indicators, and fixed/random-effects models chosen by heterogeneity.

**Results:**

A total of 22 studies involving 1,872 patients (17 high-quality and 5 moderate-quality) were included. High PD-L1 expression was significantly associated with higher tumor grade (OR = 3.78, 95% CI:2.04-7.01; p<0.001), poorer differentiation (OR = 2.80, 95% CI:1.18-6.65; p=0.020), increased PD-1 expression (OR = 4.15, 95% CI:2.16-7.99; p<0.001), and shorter overall survival (HR = 1.66, 95% CI:1.32-2.10; p<0.001). No significant associations were found with sex, age, histological type, tumor stage, invasion, metastasis, CD8+ T cell/FOXP3+ T cell infiltration, or mismatch repair (MMR) status. No publication bias existed.

**Conclusion:**

High PD-L1 expression in GEP-NENs correlates with aggressive clinicopathological features, PD-1 upregulation, and unfavorable prognosis. PD-L1 may serve as a prognostic biomarker and therapeutic target for immune checkpoint inhibitors, particularly in high-grade/poorly differentiated tumors. Large-scale prospective studies are needed for validation.

**Systematic review registration:**

https://www.crd.york.ac.uk/PROSPERO, identifier CRD420251048602.

## 1 Introduction

Neuroendocrine neoplasms (NENs) are a highly heterogeneous group of tumors originating from the neuroendocrine system. The cells giving rise to these tumors are widely distributed throughout the body, so NENs can occur almost anywhere in the body, but they are most commonly found in the gastrointestinal tract, pancreas, or lungs (1, 2). Based on the degree of differentiation of tumor cells, NENs can be further classified into well-differentiated neuroendocrine tumors (NETs) and poorly differentiated neuroendocrine carcinomas (NECs). Of these, gastroenteropancreatic neuroendocrine neoplasms (GEP-NENs) account for approximately 70% of NENs (3). In recent years, the incidence of GEP-NENs is rising in Europe and the United States (4, 5), posing a public health challenge. Rindi et al. demonstrated that GEP-NENs can be stratified into three grades based on tumor cell morphology (well-differentiated *vs*. poorly-differentiated), along with mitotic rate and/or Ki-67 proliferation index (6). Grading has a significant impact on the development of treatment regimens. Currently, surgical resection is an effective treatment for low-grade GEP-NENs (7). However, some patients are not suitable for radical surgery due to large tumors or distant metastases, and GEP-NENs also have a high rate of postoperative recurrence (8). At this point, for patients, including high-grade GEP-NENs, chemotherapy, radiation effect therapies, biologic therapies, and other treatments are available but still have limited effectiveness (9–12). Therefore, there is an urgent need to find effective therapeutic targets to enable more targeted and personalized treatment.

Cancer immunotherapy, as an emerging therapeutic strategy, has made remarkable scientific progress in the past few years. It identifies and attacks tumor cells by activating or enhancing the body’s own immune system, and has made key breakthroughs in the field of precision targeted tumor therapy. Programmed cell death protein 1 (PD-1) and its ligand (programmed death ligand 1, PD-L1) are important immune checkpoint molecules. Binding between PD-L1 and PD-1 leads to apoptosis of activated T cells, a mechanism by which tumor cells evade host immunity and survive (13). The use of PD-1/PD-L1 inhibitors blocks the interaction between PD-1 and PD-L1, removes the braking effect of inhibitory receptors on the surface of T cells, and activates the anti-tumor immune response, a known mechanism that leads to T cell dysfunction (14, 15). Recent advances in immunotherapy have led to an increasing interest in the study of PD-L1 as a biomarker for predicting prognosis and determining a patient’s eligibility for targeted therapy.PD-L1 has been shown to be expressed in a wide variety of tumors. Therefore, PD-1/PD-L1 inhibitors have also been more widely used in a variety of cancers, such as melanoma, lung and breast cancers (16–18). Currently, several experiments have demonstrated that GEP-NENs tumor tissues can also express PD-L1, and correlations between PD-L1 and patients’ pathological features, immune markers and prognosis have been found. However, the findings of these studies are inconsistent, which greatly limits the clinical use of immunotherapy and related biomarkers in GEP-NENs. Furthermore, due to the rarity of NENs, the sample sizes in relevant clinical trials are limited, which may affect the statistical significance of research findings and the scalability of clinical applications.

Therefore, this study aims to integrate currently published clinical research data through meta-analysis to systematically analyze the potential relationship between PD-L1 expression and clinical-pathological characteristics and prognosis in GEP-NENs. It also seeks to preliminarily explore its potential application as a prognostic biomarker, provide clinical insights for specific GEP-NENs patients receiving PD-1/PD-L1 inhibitor therapy, and accumulate foundational data and theoretical references for future research in this field.

## 2 Methods

The study was conducted in strict adherence to the Preferred Reporting Items for Systematic Reviews and Meta-Analyses (PRISMA) guidelines (19). It was registered with the International Registry of Prospective Systematic Reviews (PROSPERO) under the registration number CRD420251048602 (available at: https://www.crd.york.ac.uk/prospero).

### 2.1 Search strategy

This study systematically searched multiple databases including Medline/PubMed, Web of Science, Embase, and the Cochrane Library. The search period spanned from the time of literature compilation to November 2025, with no language restrictions. Taking PubMed as an example, its MeSH terms included “Programmed cell death 1 receptor/B7-H1 antigen” and “Neuroendocrine tumor/Neuroendocrine carcinoma.” MeSH terms were combined using Boolean logic: matching terms were linked with “OR,” and results from different groups were merged using “AND” (**Supplementary Table 1**). Additionally, to maximize the capture of potentially relevant studies, authors manually searched the references of included studies and explored similar topics recommended by each database.

### 2.2 Selection criteria

The inclusion criteria of this research were formulated in strict accordance with the PICOS framework, which includes five core dimensions: Population, Intervention, Comparison, Outcome, and Study Design. The specific inclusion criteria corresponding to each dimension and the relevant exclusion criteria are detailed as follows:

Inclusion Criteria (Based on PICOS Framework): (1) Population (P): The research subjects were patients with GEP-NENs. (2) Intervention/Exposure (I): PD-L1 expression was detected in the research subjects. (3) Comparison (C): Implied in the analysis of PD-L1 expression levels (e.g., high *vs*. low expression) in relation to clinicopathological features or prognosis. (4) Outcome (O): The study provided available data on the relationship between PD-L1 expression and clinicopathological features (e.g., tumor stage, differentiation degree) or prognosis (e.g., overall survival [OS], progression-free survival [PFS]) of GEP-NENs patients. (5) Study Design (S): Original research studies, including observational studies (cohort studies, case-control studies) and interventional studies (randomized controlled trials [RCT]).

Exclusion Criteria: (1) Non-original research types: Reviews, letters to the editor, editorials, commentaries, case reports, expert opinions, and meeting abstracts. (2) Defective data quality: Studies with incomplete key data or data that cannot be extracted and sorted out effectively. (3) Duplicate or low-quality research: Studies with repetitive publication, duplicated data, or failing to meet the basic academic quality requirements (e.g., unclear research design, inappropriate statistical methods).

### 2.3 Data extractions

Data were extracted independently by two researchers, and any discrepancies that arose were agreed upon through a consultative discussion with a third researcher. The following information was extracted for all included studies: (a) first author, (b) publication year, (c) country, (d) study design, (e) study population (relevant clinical pathology and prognostic), (f) detection and evaluation of PD-L1.

### 2.4 Evaluation of research quality

Two researchers assessed the quality of the included studies using the Newcastle-Ottawa Scale (NOS) (20), and disagreements that arose were resolved through consultation with a third researcher. The tool has a total score of 9 and is divided into three bands, while a score of 7-9/4-6/0–3 corresponds to high/moderate/low quality literature, respectively.

### 2.5 Statistical analysis

The meta-analysis in this study was carried out utilizing Stata version 17.0 MP (Stata Corporation, College Station, TX, USA). For dichotomous variables, the odds ratio (OR) and its 95% confidence interval (CI) were used as effect measures; for continuous variables, the mean difference (MD) or standardized mean difference (SMD) and its 95% CI were employed. The association between PD-L1 expression and OS was assessed by the hazard ratio (HR) and its 95% CI. Heterogeneity was assessed using the Q test and the I² statistic, with I² ≤ 50% and P ≥ 0.1 being considered less heterogeneous, and a fixed-effects model was used; conversely, a random-effects model was used (21, 22). Sources of heterogeneity were explored by sensitivity analysis and subgroup analysis, and publication bias was assessed. To investigate sources of heterogeneity associated with PD-L1 assessment, we conducted a multidimensional subgroup analysis comprehensively covering four core dimensions: (1) PD-L1 antibody clones, (2) threshold definitions (1%, 5%, or others), (3) scoring systems (tumor proportion score [TPS] *vs*. combined positive score [CPS]), and (4) tumor compartments (tumor cells *vs*. immune cells). By presenting effect sizes, heterogeneity indices (I²), and statistical significance across these dimensions in a stratified manner, we identified key contributors to heterogeneity and clarified the impact of different PD-L1 assessment methods on outcome consistency. Publication bias was assessed subjectively by funnel plot. Due to the relatively small number of included literature, publication bias was also assessed objectively with the help of the results of Begg’s test (23) and Egger’s test (24), which were defined as having statistically significant heterogeneity with a p-value < 0.1 or an I^2^ statistic > 50%. To minimize confounding bias, multivariate HRs were prioritized for OS analysis when both univariate and multivariate HRs were reported in the included studies. For studies without reported HRs but with available Kaplan-Meier survival curves, two independent researchers extracted and digitized the survival data using Engauge Digitizer software (v12.1; http://markummitchell.github.io/engaugedigitizer/), and discrepancies were resolved by a third researcher. The HR and 95% CI were then estimated using the Jayne F Tierney Excel program file (25), and the digitization and estimation processes were cross-validated to reduce potential bias associated with indirect HR extraction. For all combined analyses, a p-value less than 0.05 indicated statistical significance.

## 3 Results

### 3.1 Screening results and basic information of the included literature

**Figure 1:**
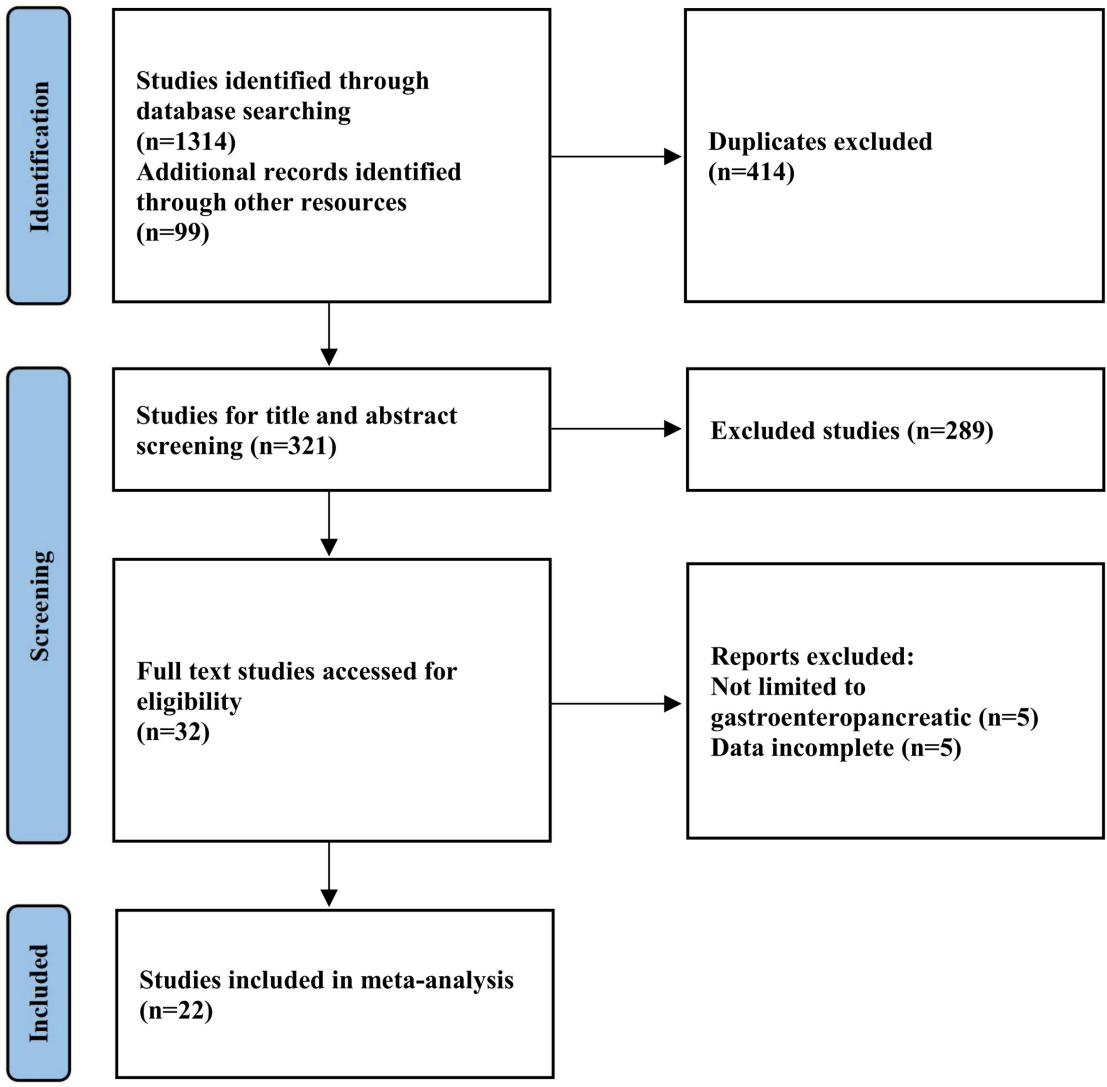
Flowchart of literature search process.

Through systematic searches of Medline/PubMed, Embase, Web of Science, and the Cochrane Library, supplemented by manual searches of recommended references, 1,413 initial articles were identified, including 414 duplicates. After removing duplicates, 321 articles remained. Following exclusion of reviews, case reports, conference papers, and 289 irrelevant articles identified through title and abstract screening, 32 articles were retained. After full-text review of the initially included studies, 10 papers that did not meet the criteria were excluded. Ultimately, 22 studies were included in the final analysis (**Figure 1**) (26–47). The basic information and relevant data of the final included studies are presented in **Table 1**.

**Table 1:** General characteristics of the 22 included studies.

Author	Year	Country	Study type	Patients (Male/Female)	Age	Tumor type	Indicators	PD-L1 cutoff definition	Outcomes	Study quality
Bösch et al.	2019 (26)	Germany	Retrospective	244 (137/107)	60 (18-92)	GEP-NENs	PD-1, PD-L1	TPS ≥ 1%	OS	7
Busico et al.	2019 (27)	Italy	Retrospective	73 (45/28)	NA	High-Grade GEP-NENs	PD-L1, MMR	≥ 1%	OS	7
Cavalcanti et al.	2017 (28)	Italy	Retrospective	57 (32/25)	56.5 (30-87)	GEP-NENs	PD-L1	Score ≥ 2	NA	6
Centonze et al.	2021 (29)	Switzerland	Retrospective	45 (28/17)	61 (33–78)	High-Grade GEP-NENs	PD-L1	NA	OS	7
Chen et al.	2024 (30)	China	Retrospective	56 (29/27)	40 (23–74)	Pan-NENs	PD-L1	≥ 5%	NA	6
Cheng et al.	2022 (31)	China	Retrospective	56 (46/10)	NA	GEJ NECs and gastric non-cardiac NECs	PD-L1	CPS ≥ 1	OS	7
Cives et al.	2019 (32)	USA	Retrospective	102 (52/50)	60 (27-95)	Small bowel NETs	PD-1, PD-L1	TPS ≥1% or ≥50%	OS	9
Gürler et al.	2024 (33)	Turkey	Retrospective	83 (49/34)	55.7 (18.4-77.9)	GEP-NENs	PD-L1	≥ 1%	NA	6
Hasegawa et al.	2020 (34)	Japan	Retrospective	20 (11/9)	62 (28-82)	GEP-NENs	PD-1, PD-L1, CD8, FOXP3	> 1%	NA	8
Kim et al.	2016 (35)	Korea	Retrospective	32 (18/14)	60 (35-88)	Metastatic GEP-NETs	PD-L1	> 1%	OS	7
Liang et al.	2025 (36)	China	Retrospective	112 (68/44)	40 (28-86)	G-NENs	PD-L1	H-score > median	NA	7
Milione et al.	2019 (37)	Italy	Retrospective	350 (NA)	NA	GEP-NENs	PD-1, PD-L1, CD8	Specific level > 0	OS	9
Multone et al.	2024 (38)	Switzerland	Retrospective	68 (43/25)	65.5 (19-83)	NECs of the digestive system	PD-L1, MMR, TILs (CD3)	TPS > 1% and/or CPS > 1	NA	8
Oktay et al.	2019 (39)	Turkey	Retrospective	59 (30/29)	NA	GEP-NENs	PD-L1	NA	NA	7
Ono et al.	2018 (40)	Japan	Retrospective	136 (82/54)	65.5 (24-85)	Gastrointestinal NENs	PD-L1	Specific level > 0	NA	6
Roberts et al.	2017 (41)	USA	Retrospective	37 (18/19)	62 (32-80)	NECs of the digestive system	PD-1, PD-L1	score ≥ 1	NA	7
Rosery et al.	2021 (42)	Germany	Retrospective	37 (19/18)	61.7 (29-80)	G3 GEP-NEN	PD-1, PD-L1, TILs (CD8)	CPS ≥ 1	OS	8
Wang et al.	2019 (43)	China	Retrospective	120 (80/40)	55.2 (12-84)	GEP-NENs	PD-1, PD-L1	Specific score ≥ 3	mOS	7
Xing et al.	2020 (44)	China	Retrospective	31 (20/11)	65 (20-81)	NECs of the digestive system	PD-L1	TPS > 1%	OS	7
Yamashita et al.	2020 (45)	Japan	Retrospective	25 (21/4)	73 (52–86)	G-NECs	PD-L1	CPS ≥ 1	OS	8
Yang et al.	2019 (46)	China	Retrospective	43 (35/8)	62 (33-82)	G-NECs	PD-1, PD-L1, TILs (CD8 and FOXP3)	Composite score ≥ 4	OS	8
Yao et al.	2021 (47)	USA	Retrospective	86 (51/35)	NA (33–85)	GEP-NENs	PD-L1, TILs (CD8)	> 1%	NA	6

NA, not available; GEP-NENs, gastroenteropancreatic neuroendocrine neoplasms; Pan-NENs, pancreatic neuroendocrine neoplasms; GEJ NECs, gastroesophageal junctiona neuroendocrine carcinomas; NETs, neuroendocrine tumors; G-NENs, gastric neuroendocrine neoplasms; PD-1, programmed cell death protein 1; PD-L1, programmed death ligand 1; TILs, tumor-infiltrating lymphocytes; MMR, mismatch repair protein; TPS, tumor proportion score; CPS, combined positive score; OS, overall survival.

### 3.2 Summary of quality and risk of bias of included studies

The authors assessed the quality of the included studies using the NOS scale (**Supplementary Table 2**). Results indicated that 17 studies were of high quality, 5 were of moderate quality, and none were of low quality. This suggests that the overall quality of the 22 included studies was relatively high, enhancing the reliability of the meta-analysis findings.

### 3.3 Correlation between PD-L1 expression and clinicopathologic features of patients with GEP-NENs

**Figure 2:**
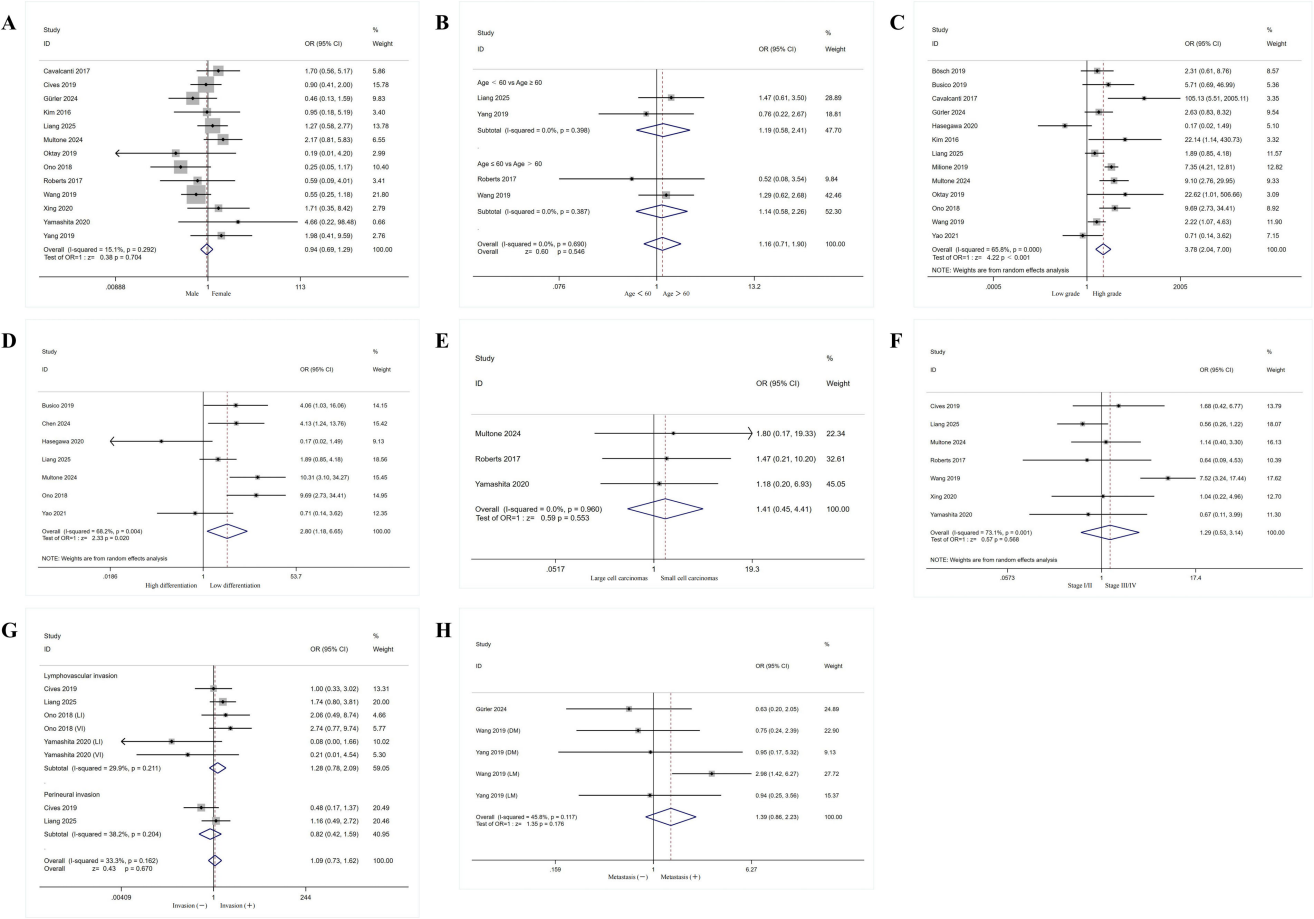
Forest plot showing the correlation between PD-L1 expression and clinical pathology in patients with gastroenteropancreatic neuroendocrine neoplasms. **(A)** Gender, **(B)** Age, **(C)** Grade, **(D)** Histological differentiation, **(E)** Pathology, **(F)** Stage, **(G)** Invasion, **(H)** Metastasis. DM, distant metastasis; LI, lymphatic invasion; LM, lymphatic metastasis; VI, venous invasion.

The authors further explored the relationship between PD-L1 expression and characteristics (such as gender, age, grade, histological differentiation, pathology, stage, invasion and metastasis) in patients with GEP-NENs (**Figure 2**, **Table 2**).

**Table 2:** Summary of the combined effect sizes for each parameter.

Outcomes	No. of cohorts	No. of patients	OR (95% CIs) or HR (95% CIs)	P-value	Heterogeneity test		Egger’s Test (P-value)
					I^2^	P-value	
Clinicopathologic features							
Gender	13	905	0.94 (0.69, 1.29)	0.704	15.10%	0.292	0.443
Age	4	312	1.16 (0.71, 1.90)	0.546	0.00%	0.690	0.144
Grade	13	1415	3.78 (2.04, 7.00)	**< 0.001**	65.80%	< 0.001	0.127
Histological differentiation	7	537	2.80 (1.18, 6.65)	**0.020**	68.20%	0.004	0.895
Pathology	3	98	1.41 (0.45, 4.41)	0.553	0.00%	0.960	0.145
Stage	7	491	1.29 (0.53, 3.14)	0.568	73.10%	0.001	0.475
Invasion	6	649	1.09 (0.73, 1.62)	0.670	33.30%	0.162	0.655
Metastasis	5	409	1.39 (0.86, 2.23)	0.176	45.80%	0.117	0.072
Immune markers							
PD-1 expression	5	352	4.15 (2.16, 7.99)	**< 0.001**	0.00%	0.891	0.809
CD8 expression	4	131	1.36 (0.66, 2.82)	0.403	28.10%	0.243	0.247
FOXP3 expression	2	63	1.21 (0.42, 3.46)	0.724	0.00%	0.347	–
MMR status	2	93	0.57 (0.15, 2.18)	0.415	0.00%	0.718	–
Prognosis							
OS	11	805	1.66 (1.32, 2.10)	**< 0.001**	47.40%	0.040	0.975

PD-1, programmed cell death protein 1; MMR, mismatch repair protein; OS, overall survival. Bold values indicate statistically significant associations between PD-L1 expression and the corresponding outcome (p < 0.05).

Thirteen cohorts (28, 32, 33, 35, 36, 38–41) examined the relationship between PD-L1 expression and patient gender. Results indicated no heterogeneity among included studies (I²=15.1%, P = 0.292). Using a fixed-effect model yielded an OR = 0.94, 95% CI: 0.69-1.29, suggesting no association between PD-L1 expression and gender (P = 0.704). Four cohorts (36, 41, 43, 46) examined the relationship between PD-L1 expression and patient age. Results indicated no heterogeneity among included studies (I²=0.00%, P = 0.690). Using a fixed-effect model yielded an OR of 1.16 (95% CI: 0.71-1.90), suggesting no association between PD-L1 expression and age (P = 0.546).

Thirteen cohorts (26–28, 33–40, 43, 47) examined the relationship between PD-L1 expression and tumor grade (grouping G1 and G2 together versus G3 [including well-differentiated NET G3] and poorly differentiated NEC). Results showed significant heterogeneity among included studies (I² = 65.80%, P < 0.001). Using a random-effects model yielded an OR of 3.78 (95% CI: 2.04-7.00), indicating a significant association between PD-L1 expression and high tumor grade (P < 0.001). Seven cohorts (27, 30, 34, 36, 38, 40, 47) examined the relationship between PD-L1 expression and tumor histological differentiation (with NET and NEC grouped separately). Results indicated heterogeneity among included studies (I² = 68.20%, P = 0.004). Using a random-effects model yielded an OR of 2.80 (95% CI: 1.18-6.65), confirming a significant association between PD-L1 expression and poorly differentiated tumors (P = 0.020).

Three cohorts (38, 41, 45) examined the relationship between PD-L1 expression and tumor histological type (with small cell carcinomas as one group and large cell carcinomas as another). Results showed no heterogeneity among included studies (I²=0.00%, P = 0.960). Using a fixed-effect model yielded an OR of 1.41 (95% CI: 0.45-4.41), indicating no association between PD-L1 expression and pathological type (P = 0.553). Seven cohorts (32, 36, 38, 41, 43–45) examined the relationship between PD-L1 expression and tumor stage (grouped as Stage I/II versus Stage III/IV). Results indicated significant heterogeneity among included studies (I² = 73.10%, P = 0.001). Using a random-effects model yielded an OR of 1.29 (95% CI: 0.53-3.14), suggesting no association between PD-L1 expression and tumor stage (P = 0.568).

Six cohorts (32, 36, 40, 45) examined the relationship between PD-L1 expression and tumor invasion. Results showed no heterogeneity among included studies (I²=33.300%, P = 0.162). Using a fixed-effect model yielded an OR = 1.09, 95% CI: 0.73-1.62, indicating no association between PD-L1 expression and tumor invasion (P = 0.670). Five cohorts (33, 43, 46) examined the relationship between PD-L1 expression and tumor metastasis. Results indicated no heterogeneity among the included studies (I²=45.80%, P = 0.117). Using a fixed-effect model yielded an OR of 1.39 (95% CI: 0.86-2.23), suggesting no association between PD-L1 expression and tumor metastasis (P = 0.176).

### 3.4 Correlation of PD-L1 expression with immune markers in patients with GEP-NENs

**Figure 3:**
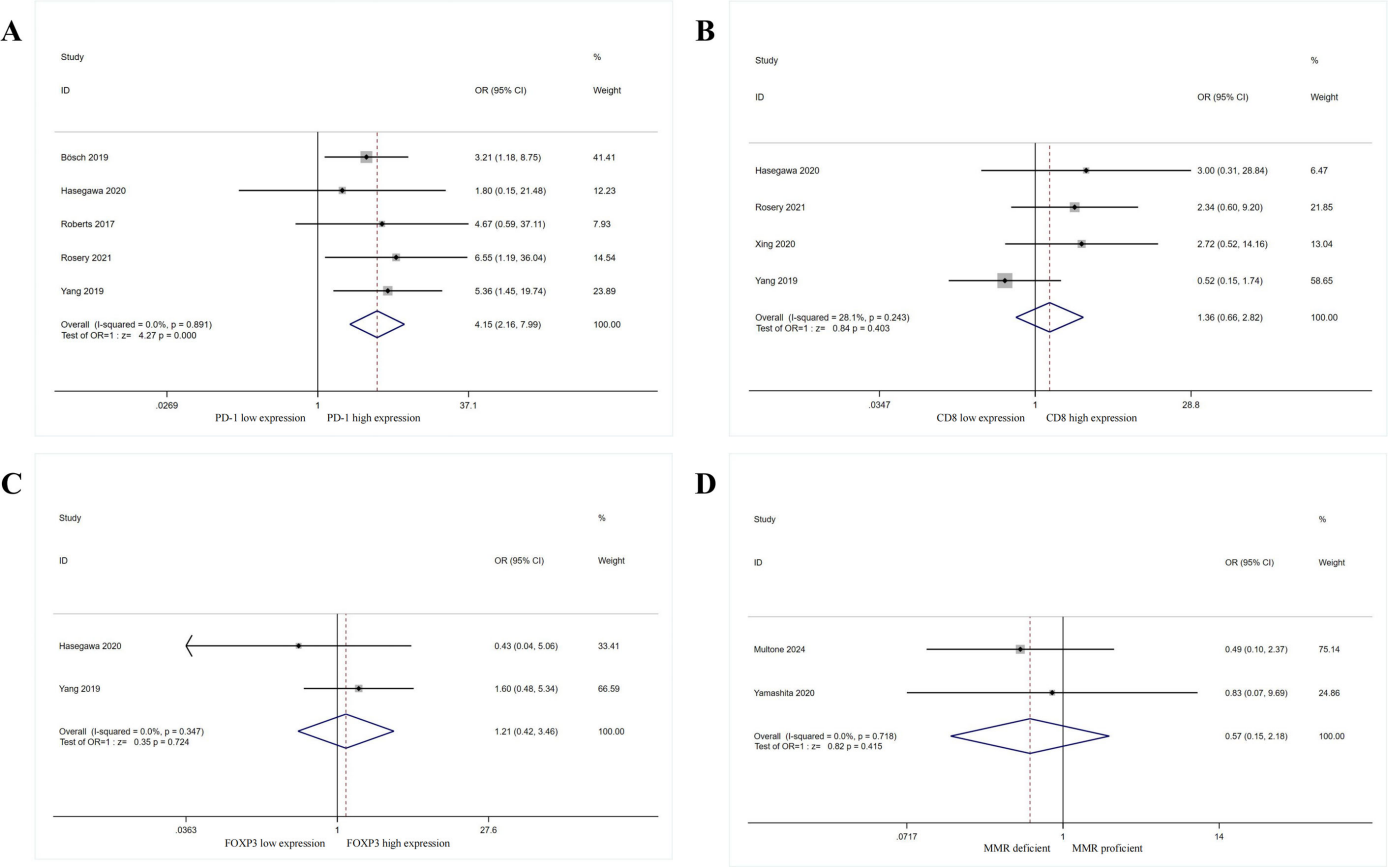
Forest plot showing the correlation between PD-L1 expression and immunobiological markers in patients with gastroenteropancreatic neuroendocrine neoplasms. **(A)** PD-1 expression, **(B)** CD8 expression, **(C)** FOXP3 expression, **(D)** MMR status.

The authors further explored the relationship between PD-L1 expression and immune markers (such as PD-1, CD8+ TILs, Foxp3, MMR) in patients with GEP-NENs (**Figure 3**, **Table 2**).

Five cohorts (26, 34, 41, 42, 46) examined the relationship between PD-L1 expression and PD-1 expression in patients with GEP-NENs. Results showed no heterogeneity among included studies (I²=0.00%, P = 0.891). Using a fixed-effect model yielded an OR of 4.15 (95% CI: 2.16-7.99), indicating a significant correlation between PD-L1 expression and PD-1 expression (P<0.001). Four cohorts (34, 42, 44, 46) examined the relationship between PD-L1 expression and CD8 expression in patients. Results showed no heterogeneity among included studies (I²=28.10%, P = 0.243). Using a fixed-effect model yielded an OR = 1.36, 95% CI: 0.66-2.82, indicating no correlation between PD-L1 expression and CD8 expression (P = 0.403). Two cohorts (34, 46) examined the relationship between PD-L1 expression and patient FOXP3 expression. Results showed no heterogeneity among included studies (I²=0.00%, P = 0.347). Using a fixed-effect model yielded an OR = 1.21, 95% CI: 0.42-3.46, indicating no association between PD-L1 expression and FOXP3 expression (P = 0.724). However, due to the small number of included studies (n=2), this conclusion is preliminary and lacks sufficient statistical power; validation with larger, multicenter cohorts is required. Two cohorts (38, 45) examined the relationship between PD-L1 expression and patient MMR status. Results showed no heterogeneity among included studies (I²=0.00%, P = 0.718). Using a fixed-effect model yielded an OR of 0.57 (95% CI: 0.15-2.18), indicating no association between PD-L1 expression and MMR status (P = 0.415). Similarly, the small sample size (n=2) limits the generalizability of this finding, and further studies are needed to confirm this result.

### 3.5 Correlation of PD-L1 expression with OS in patients with GEP-NENs

**Figure 4:**
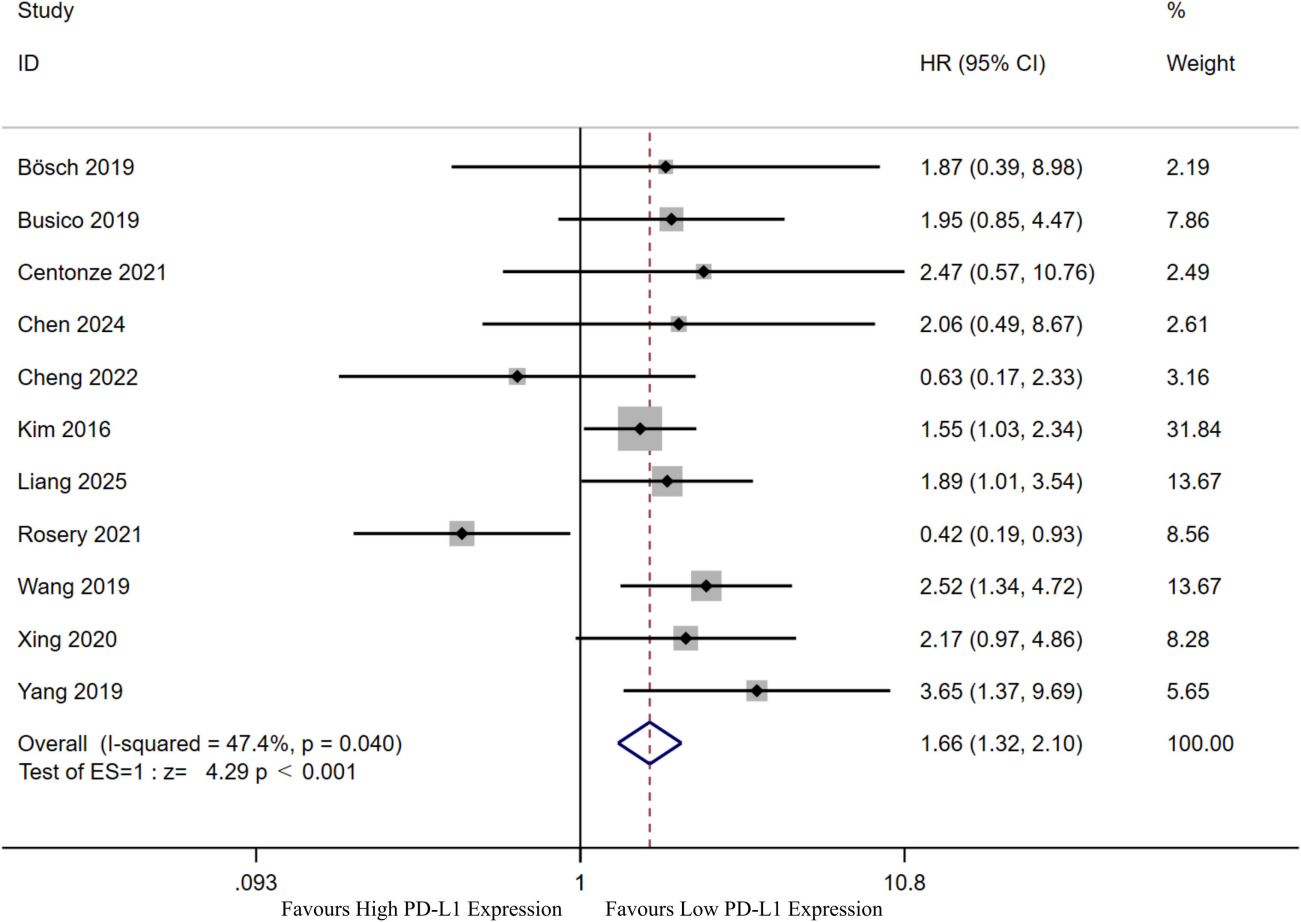
Forest plot showing the correlation between PD-L1 expression and prognosis (overall survival) in patients with gastroenteropancreatic neuroendocrine neoplasms.

The authors further explored the relationship between PD-L1 expression and OS in patients with GEP-NENs (**Figure 4**, **Table 2**). Eleven cohorts (26, 27, 30, 31, 35, 36 42–44, 46) examined the relationship between PD-L1 expression and OS in patients. Results demonstrated heterogeneity among included studies (I²=47.40%, P = 0.040). Using a random-effects model yielded an HR of 1.66 (95% CI: 1.32-2.10), indicating a significant association between PD-L1 expression and OS (P<0.001).

### 3.6 Subgroup analysis

This study conducted a multidimensional subgroup analysis targeting key heterogeneity factors associated with PD-L1 assessment (**Supplementary Tables 3**, **4**). Results revealed that within the PD-L1 antibody clonal subgroups, SP142 (OR = 8.99, 95% CI:1.61-50.15, P = 0.012), SP263 (OR = 4.84, 95% CI:1.43-16.41, P = 0.011), 28-8 (OR = 1.97, 95% CI:1.03-3.74, P = 0.039), and 22C3 (OR = 7.35, 95% CI:4.21-12.81, P<0.001) clones showed significant associations with treatment efficacy. In contrast, E1L3N (OR = 12.72, 95% CI:0.23-706.10, P = 0.215) and NAT105 (OR = 0.17, 95% CI:0.02-1.49, P = 0.109) showed no significant association. Regarding heterogeneity, E1L3N cloning exhibited high heterogeneity (I²=84.0%, P = 0.013), SP263 showed moderate heterogeneity (I²=53.9%, P = 0.141), while SP142 and 28–8 exhibited low heterogeneity; In PD-L1 cutoff-defined subgroups, the “Other” category cutoff demonstrated the strongest therapeutic effect association (OR = 5.40, 95% CI:2.53-11.53, P<0.001) showed the most significant association with treatment effect. Although the 1% cutoff group did not show a significant association (OR = 1.87, 95% CI:0.67-5.21, P = 0.231), it demonstrated significant benefit in OS analysis (HR = 1.71, 95% CI:1.23-2.38, P = 0.001). Heterogeneity was moderate in the 1% cutoff group (I²=51.5%, P = 0.067), while the “Other” cutoff group exhibited high heterogeneity (I²=72.6%, P = 0.003). In the PD-L1-detected tumor region subgroup, combined detection of tumor cells and immune cells (OR = 4.63, 95% CI:2.06-10.41, P<0.001) showed the most significant association with treatment response. Neither the tumor cell-only detection group (OR = 2.60, 95% CI:0.80-8.46, P = 0.113) nor the immune cell-only detection group (OR = 5.71, 95% CI:0.69-46.99, P = 0.105) did not reach statistical significance. Both the tumor cell detection group (I²=68.8%, P = 0.012) and the combined detection group (I²=68.6%, P = 0.004) exhibited high heterogeneity. Within PD-L1 scoring system subgroups, the combined CPS/TPS scoring system (OR = 9.10, 95% CI:2.76-29.95, P<0.001) showed the most significant association with treatment efficacy, while TPS alone (OR = 2.31, 95% CI:0.61-8.76, P = 0.220) showed no significant association but demonstrated significant benefit in OS analysis (HR = 2.10, 95% CI: 1.02-4.31, P = 0.043). CPS score alone demonstrated significant protective effects in OS analysis (HR = 0.47, 95% CI: 0.24-0.92, P = 0.029).

### 3.7 Publication bias

Funnel plots visually demonstrate that no significant publication bias exists across the pooled effect sizes (**Supplementary Figure 1**). Furthermore, quantitative assessments of publication bias using Begg’s test and Egger’s test reveal no evidence of significant publication bias (**Table 2**).

### 3.8 Sensitivity analysis

Sensitivity analysis revealed that excluding the study by Wang et al. (43) significantly altered the pooled OR to 0.79 (95% CI: 0.36-1.73), which falls outside the 95% confidence interval of the original pooled result (**Supplementary Figure 2H**). This indicates significant instability of the metastasis-related finding, and due to the lack of robustness, this result should be interpreted with extreme caution. When examining other indicators, removing individual studies did not result in significant changes to the pooled effect sizes, suggesting the stability and reliability of the meta-analysis results (**Supplementary Figure 2**).

## 4 Discussion

This systematic review and meta-analysis aimed to investigate the correlation between PD-L1 expression and the clinical-pathological characteristics, immune markers (such as PD-1 expression), and prognosis of GEP-NENs. Our primary findings are summarized as follows: (1) High PD-L1 expression was significantly associated with higher WHO tumor grades, including well-differentiated grade 3 NET (NET G3) and poorly differentiated NEC); (2) High PD-L1 expression correlated with PD-1 expression in the tumor microenvironment; (3) High PD-L1 expression predicted poorer OS. These findings suggest PD-L1 may serve as an independent prognostic marker for GEP-NENs and further propose the hypothesis that immune checkpoint inhibitors (ICIs), such as PD-1/PD-L1 inhibitors, may demonstrate therapeutic efficacy in high-grade GEP-NENs. However, a critical limitation should be highlighted upfront: PFS—a key prognostic indicator for GEP-NENs that reflects disease progression and treatment response—was not analyzed in this meta-analysis due to insufficient data reporting in included studies, which limits the comprehensiveness of our prognostic assessment. Most included studies were retrospective, and the lack of direct correlation between PD-L1 expression and ICI efficacy metrics (such as objective response rate [ORR] and PFS) further limits the applicability of these findings to clinical treatment decisions.”

In malignant tumors, PD-L1 expression is commonly regarded as a poor prognostic factor (48) and is closely associated with key clinical-pathological features such as tumor grading (49, 50). Consistent with these findings, our study confirms that high PD-L1 expression in GEP-NENs is significantly correlated with higher WHO grades (including NET G3 and NEC), potentially reflecting increased tumor invasiveness and immune evasion mechanisms. This conclusion is supported by existing research: interferon-γ (IFN-γ)-induced PD-L1 expression in tumor cells is a key mechanism of adaptive immune resistance, suppressing T-cell activity. Notably, PD-L1 expression is more pronounced in tumors with higher malignancy (accompanied by stronger immune pressure) (51). Furthermore, high PD-L1 expression in high-grade GEP-NENs frequently accompanies adverse pathological features (e.g., high Ki67 index) and an immunosuppressive tumor microenvironment (52, 53). Luo et al. demonstrated that miR-23a/27a/24 cluster upregulation correlates with tumor immune evasion and PD-L1 overexpression, a phenomenon more pronounced in patients with higher malignancy and poorer prognosis (54). Yang et al. demonstrated that activation of the mitogen-activated protein kinase (MAPK) pathway—commonly observed in aggressive tumors—directly induces PD-L1 accumulation on the cell surface, thereby accelerating immune evasion and drug resistance development (55). Notably, PD-L1 expression patterns may vary across GEP-NENs subtypes. For instance, in pancreatic NETs, PD-L1 expression correlates with differentiation grade, with higher-grade tumors more likely to be PD-L1-positive, suggesting tumor heterogeneity may influence immune checkpoint expression (56, 57).

This meta-analysis further confirms that high PD-L1 expression in GEP-NENs correlates with PD-1 expression and poorer OS, highlighting PD-L1’s pivotal role in mediating tumor immune escape. The PD-1/PD-L1 pathway is central to this process: PD-L1 expressed by tumor or immune cells binds to PD-1 on T-cell surfaces, suppressing antitumor function and inducing T-cell exhaustion. The co-occurrence of high PD-L1 and PD-1 expression observed in this study directly validates the activity of this pathway in GEP-NENs. This finding aligns with previous research linking PD-L1 positivity to T cell exhaustion (e.g., CD8+ T cell dysfunction) and poor prognosis (53, 58). This also suggests that PD-L1 expression in GEP-NENs may reflect an “immunohot” yet simultaneously immunosuppressive tumor microenvironment (characterized by abundant tumor-infiltrating lymphocytes), thereby impacting patient survival (58). Importantly, the association between PD-L1 positivity and poor OS has been widely reported across multiple cancers, including hepatocellular carcinoma (59), gastric cancer (60), and urothelial carcinoma (61). In GEP-NENs, this correlation may be amplified due to tumor biological heterogeneity; for example, PD-L1 expression in high-grade tumors correlates closely with metastasis and drug resistance (62). Collectively, these findings not only confirm high PD-L1 expression as a potential prognostic marker for GEP-NENs but also suggest that patients with high-grade GEP-NENs may benefit from immunotherapy, consistent with existing research evidence.

Beyond its prognostic value, PD-L1 status is a recognized predictor of ICI efficacy across multiple cancer types (61, 63), and emerging data also suggest that patients with high-grade GEP-NENs may have therapeutic response potential to PD-1/PD-L1 inhibitors. For example, response rates to dual CTLA-4/PD-1 targeting range from 9% to 44% (64, 65), and PD-L1-positive tumors demonstrate higher sensitivity to agents such as avelumab (66). However, it is important to note that the overall response rate to ICIs in NEN patients remains generally low, indicating that PD-L1 is not the sole determinant of treatment efficacy. Characteristics of the tumor immune microenvironment, such as tumor-infiltrating lymphocyte density, also play a significant role (67). Therefore, combining PD-L1 with other biomarkers may further optimize prognostic assessment and treatment prediction models for GEP-NENs.

To address the variability in PD-L1 assessment methods across studies, this research conducted a multidimensional subgroup analysis targeting key heterogeneity factors in PD-L1 evaluation, including antibody clones, cutoff definitions, tumor area detection, and scoring systems. Results revealed that antibody clones SP142, 28-8, 22C3, and SP263; the “other” category cutoff versus the traditional 1% cutoff (for OS assessment); combined detection of tumor cells and immune cells versus tumor cell-only detection (for OS prediction); and CPS/TPS versus standalone TPS/CPS scoring demonstrated superior correlations with tumor grade, histological differentiation, PD-1 expression, and OS. Furthermore, the analysis identified the E1L3N antibody, the “Other” cutoff group, and the tumor cell/combined detection group as primary sources of heterogeneity, offering optimized detection strategies for different clinical scenarios. These findings support standardized PD-L1 testing (prioritizing high-quality antibodies, combined detection, or customized scoring systems) and personalized decision-making (adjusting cutoffs based on disease characteristics). However, limitations such as data gaps, high heterogeneity, and uncontrolled confounders in certain subgroups should be acknowledged. These issues also underpin the study limitations discussed later. Future efforts should focus on expanding sample sizes, establishing unified testing standards, and integrating other immunobiomarkers for combined assessment to enhance the precision of identifying patients likely to benefit from immunotherapy.

In summary, our findings confirm that PD-L1 expression in GEP-NENs correlates positively with high WHO grading (NET G3/NEC) and PD-1 expression. These results support the hypothesis that this patient subgroup relies heavily on the PD-1/PD-L1 pathway for immune evasion and may therefore benefit from anti-PD-1/PD-L1 therapy. In clinical practice, PD-L1 testing can be combined with WHO grading and molecular features (e.g., DLL3 mutation) for patient stratification (52, 57). Prospective exploration of ICIs or combination therapies (e.g., avelumab + anti-angiogenic agents) warrants consideration in high PD-L1-positive cases (64, 66). Future research on GEP-NENs should focus on four key directions (classified by research objectives): (1) Validation: Multicenter studies to validate the association between PD-L1 expression and clinical-pathological features, immune markers, and prognosis; (2) Mechanistic exploration: Elucidate PD-L1 regulatory mechanisms through multi-omics analysis (68) and clarify the molecular role of the PD-1/PD-L1 pathway in GEP-NENs pathogenesis; (3) Clinical translation: Conduct PD-L1-guided RCTs (69), systematically evaluate the clinical efficacy, safety, and predictive value of PD-1/PD-L1 pathway-targeted antibodies through prospective trials (establishing their association with ORR and PFS), and validate the hypothesis that PD-L1 expression guides immunotherapy selection in high-grade GEP-NENs; (4) Methodological Standardization: Develop standardized PD-L1 detection guidelines to reduce heterogeneity. Furthermore, future studies should adopt more rigorous designs, expand sample sizes, standardize survival analysis methods, extend follow-up periods, and increase implementation of high-quality clinical research such as RCTs to enrich data dimensions, reduce study bias, and enhance the reliability of conclusions.

Although this meta-analysis provides compelling evidence, several limitations exist: (1) Most notably, lack of standardization in PD-L1 assessment across included studies, including variations in antibody clones (e.g., 22C3, SP263), cutoff values (TPS ranging from 1% to 5%), scoring systems (TPS *vs*. CPS), and tumor regions (tumor cells *vs*. immune cells). This heterogeneity may have compromised the consistency of results and hindered direct comparisons between studies. Furthermore, the small sample sizes in each subgroup significantly impaired the quality of subgroup analyses, substantially reducing the power to assess the impact of these factors on heterogeneity. Therefore, standardizing PD-L1 testing in GEP-NENs is urgently needed to enhance the reliability of its clinical application. (2) Despite including 22 studies, key subgroup analyses (e.g., FOXP3, MMR status, histopathological subtype) were based on only 2–3 studies, resulting in insufficient statistical power. Conclusions remain preliminary and require further validation. Furthermore, the association between PD-L1 expression and tumor metastasis is unstable: sensitivity analysis revealed significant shifts in the pooled effect size after excluding one study, indicating dependence on individual studies and potential confounding by the proportion of high-grade/advanced patients. (3) Some studies lacked raw survival data, necessitating indirect estimation of HRs from Kaplan-Meier curves; This indirect method may introduce errors, compromising the validity of pooled results. (4) Correlation does not imply causation: This analysis confirms only an association between PD-L1 expression and prognosis, not causality. Potential biological mechanisms require further experimental validation. (5) Study design limitations: Research on GEP-NENs remains scarce and predominantly retrospective. Existing prognostic studies predominantly focus on OS while neglecting critical indicators like PFS. This omission prevents a comprehensive evaluation of PD-L1’s prognostic value, as PFS often complements OS by capturing early treatment responses that OS may not reflect in GEP-NENs. Research on staging for GEP-NETs and GEP-NECs is also scarce. Furthermore, inconsistencies in study focus (e.g., age groups, primary tumor sites) limited literature inclusion, resulting in high heterogeneity among enrolled studies. Additionally, prognostic analyses failed to effectively distinguish between GEP-NETs and GEP-NECs, further compromising result accuracy.

## 5 Conclusion

This meta-analysis demonstrated that PD-L1 is aberrantly expressed in GEP-NENs and significantly correlated with tumor grade, PD-1 expression, and OS. Therefore, PD-L1 holds promise as a highly prospective prognostic biomarker and potential therapeutic target for immunotherapy interventions in GEP-NENs, warranting further in-depth investigation. Future studies should focus on elucidating the underlying molecular mechanisms of the PD-1/PD-L1 pathway in GEP-NENs pathogenesis and systematically assessing the clinical efficacy, safety, and predictive value of antibody-based therapies targeting this pathway. Notably, due to the inherent limitations of the current meta-analysis (e.g., heterogeneity among included studies, potential publication bias), large-scale, multicenter prospective cohort studies and mechanistic explorations using robust preclinical models are imperative to validate and extend these findings. Such efforts will provide more robust evidence to support the optimization of precision medicine strategies for GEP-NENs, ultimately facilitating the translation of scientific insights into improved clinical outcomes for patients with this disease.

## Data Availability

The original contributions presented in the study are included in the article/**Supplementary Material**. Further inquiries can be directed to the corresponding authors.
