# Supplementary material for: Correlation between PD-L1 expression and clinical pathology, immunobiological markers, and prognosis in gastroenteropancreatic neuroendocrine neoplasms: a systematic review and meta-analysis
Source: Front Immunol. 2026 Feb 12;17:1772011. doi: 10.3389/fimmu.2026.1772011 (PMC12935982; doi:10.3389/fimmu.2026.1772011)
Supplement: Supplementary file 1 [file DataSheet1.docx]

**Supplementary Files**

**Contents**

**Supplementary Table 1 – Search Strategy ………………………………………………………………………………………………… 2**

**Supplementary Table 2 – The quality assessment of included studies …………………………………………………………………… 3**

**Supplementary Table 3 – The information on PD-L1 antibodies of included studies …………………………………………………… 5**

**Supplementary Table 4 – Subgroup analysis of selected pooled-results based on PD-L1 information ………………………………… 7**

**Supplementary Figure 1 – Funnel plot …………………………………………………………………………………………………… 11**

**Supplementary Figure 2 – Sensitivity analysis …………………………………………………………………………………………… 12**

**sTable 1.** A specific literature search formula using the PubMed database as an example.

| ((((((Neuroendocrine Tumors[Title/Abstract])) OR (Neuroendocrine Tumor[Title/Abstract])) OR (Tumor, Neuroendocrine[Title/Abstract])) OR (Tumors, Neuroendocrine[Title/Abstract])) OR (((((Carcinoma, Neuroendocrine[Title/Abstract])) OR (Carcinomas, Neuroendocrine[Title/Abstract])) OR (Neuroendocrine Carcinoma[Title/Abstract])) OR (Neuroendocrine Carcinomas[Title/Abstract]))) AND ((((((((((((((((((((B7-H1 Antigen[Title/Abstract])) OR (Antigen, B7-H1[Title/Abstract])) OR (B7 H1 Antigen[Title/Abstract])) OR (Programmed Death Ligand 1[Title/Abstract])) OR (Antigens, CD274[Title/Abstract])) OR (CD274 Antigens[Title/Abstract])) OR (CD274 Antigen[Title/Abstract])) OR (Antigen, CD274[Title/Abstract])) OR (B7-H1 Immune Costimulatory Protein[Title/Abstract])) OR (B7 H1 Immune Costimulatory Protein[Title/Abstract])) OR (B7H1 Immune Costimulatory Protein[Title/Abstract])) OR (PD-L1 Costimulatory Protein[Title/Abstract])) OR (Costimulatory Protein, PD-L1[Title/Abstract])) OR (PD L1 Costimulatory Protein[Title/Abstract])) OR (Programmed Cell Death 1 Ligand 1 Protein[Title/Abstract])) OR (PD-L1 Protein[Title/Abstract])) OR (PD L1 Protein[Title/Abstract])) OR (Programmed Cell Death 1 Ligand 1[Title/Abstract])) OR (((((((((((((((Programmed Cell Death 1 Receptor[Title/Abstract])) OR (PD-1 Protein[Title/Abstract])) OR (PD 1 Protein[Title/Abstract])) OR (PD-1 Receptor[Title/Abstract])) OR (PD 1 Receptor[Title/Abstract])) OR (Receptor, PD-1[Title/Abstract])) OR (Antigens, CD279[Title/Abstract])) OR (CD279 Antigens[Title/Abstract])) OR (CD279 Antigen[Title/Abstract])) OR (Antigen, CD279[Title/Abstract])) OR (PD1 Receptor[Title/Abstract])) OR (Receptor, PD1[Title/Abstract])) OR (Programmed Cell Death Protein 1[Title/Abstract])) OR (Programmed Cell Death 1 Protein[Title/Abstract]))) |
| --- |

**sTable 2.** The quality assessment of included studies.

| **Author (Year)** | **Selection** | | | | **Comparability** | **Outcomes** | | | **Total  NOS score** |
| --- | --- | --- | --- | --- | --- | --- | --- | --- | --- |
|  |  |  |  |  |  |  |  |  |  |
|  |  |  |  |  |  |  |  |  |  |
|  | **Representa-tiveness of exposed** | **Selection  of non-exposed** | **Ascertain-  ment of exposure** | **Demonstration  that outcome  of interest was not present at  the start of the study** | **Comparability  of cohorts on  basis of the design or analysis** | **Assessment  of outcome** | **Was follow-up long  enough for  outcomes  to occur** | **Adequacy of follow-up of  cohorts** |  |
|  |  |  |  |  |  |  |  |  |  |
|  |  |  |  |  |  |  |  |  |  |
|  |  |  |  |  |  |  |  |  |  |
|  |  |  |  |  |  |  |  |  |  |
|  |  |  |  |  |  |  |  |  |  |
|  |  |  |  |  |  |  |  |  |  |
| Bösch et al. (2019) | ★ | ★ | ★ |  | ★★ | ★ | ★ |  | 7 |
| Busico et al. (2019) | ★ | ★ | ★ |  | ★★ | ★ | ★ |  | 7 |
| Cavalcanti et al. (2017) | ★ | ★ | ★ |  | ★ | ★ | ★ |  | 6 |
| Centonze et al. (2021) | ★ | ★ | ★ |  | ★★ | ★ | ★ |  | 7 |
| Chen et al. (2024) | ★ | ★ | ★ |  | ★ | ★ | ★ |  | 6 |
| Cheng et al. (2022) | ★ | ★ | ★ |  | ★★ | ★ | ★ |  | 7 |
| Cives et al. (2019) | ★ | ★ | ★ | ★ | ★★ | ★ | ★ | ★ | 9 |
| Gürler et al. (2024) | ★ | ★ | ★ |  | ★ | ★ | ★ |  | 6 |
| Hasegawa et al. (2020) | ★ | ★ | ★ |  | ★★ | ★ | ★ | ★ | 8 |
| Kim et al. (2016) | ★ | ★ | ★ |  | ★★ | ★ | ★ |  | 7 |
| Liang et al. (2025) | ★ | ★ | ★ |  | ★★ | ★ | ★ |  | 7 |
| Milione et al. (2019) | ★ | ★ | ★ | ★ | ★★ | ★ | ★ | ★ | 9 |
| Multone et al. (2024) | ★ | ★ | ★ | ★ | ★★ | ★ | ★ |  | 8 |
| Oktay et al. (2019) | ★ | ★ |  |  | ★★ | ★ | ★ | ★ | 7 |
| Ono et al. (2018) | ★ | ★ | ★ |  | ★ | ★ | ★ |  | 6 |
| Roberts et al. (2017) | ★ | ★ |  |  | ★★ | ★ | ★ | ★ | 7 |
| Rosery et al. (2021) | ★ | ★ | ★ |  | ★★ | ★ | ★ | ★ | 8 |
| Wang et al. (2019) | ★ | ★ | ★ |  | ★★ | ★ | ★ |  | 7 |
| Xing et al. (2020) | ★ | ★ | ★ |  | ★★ | ★ | ★ |  | 7 |
| Yamashita et al. (2020) | ★ | ★ | ★ |  | ★★ | ★ | ★ | ★ | 8 |
| Yang et al. (2019) | ★ | ★ | ★ |  | ★★ | ★ | ★ | ★ | 8 |
| Yao et al. (2021) | ★ | ★ | ★ |  | ★ | ★ | ★ |  | 6 |

**sTable 3.** The information on PD-L1 antibodies of included studies.

| **Author (Year)** | **PD-L1 antibody manufacturer** | **PD-L1 antibody clone** | **PD-L1 antibody dilution** | **PD-L1 cutoff definition** | **Tumor compartments for PD-L1 testing** | **PD-L1 scoring system** |
| --- | --- | --- | --- | --- | --- | --- |
| Bösch et al. (2019) | Cell Signaling Technology | E1L3N | 1: 100 | TPS ≥ 1% | Tumor cells | TPS |
| Busico et al. (2019) | Ventana/Roche | SP142 | Prediluted | ≥ 1% | Immune cells | NA |
| Cavalcanti et al. (2017) | Cell Signaling Technology | E1L3N | 1: 600 | Score ≥ 2 | Tumor cells and immune cells | NA |
| Centonze et al. (2021) | NA | NA | NA | NA | Tumor cells and immune cells | NA |
| Chen et al. (2024) | Cell Signaling Technology | E1L3N | 1: 100 | ≥ 5% | Tumor cells and immune cells | NA |
| Cheng et al. (2022) | Dako | 22C3 | NA | CPS ≥ 1 | Tumor cells and immune cells | CPS |
| Cives et al. (2019) | Abcam | 28-8 | 1: 100 | TPS ≥1% or ≥50% | Tumor cells | TPS |
| Gürler et al. (2024) | Ventana/Roche | SP263 | Prediluted | ≥ 1% | Tumor cells and immune cells | NA |
| Hasegawa et al. (2020) | Abcam | NAT105 | 1: 100 | > 1% | Tumor cells | NA |
| Kim et al. (2016) | Ventana/Roche | SP142 | 1: 25 | > 1% | Tumor cells | NA |
| Liang et al. (2025) | Abcam | 28-8 | 1: 200 | H-score > median | Tumor cells | NA |
| Milione et al. (2019) | Dako | 22C3 | 1: 50 | Specific level > 0 | Tumor cells and immune cells | NA |
| Multone et al. (2024) | Ventana/Roche | SP263 | NA | TPS > 1% and/or CPS > 1 | Tumor cells and immune cells | CPS/TPS |
| Oktay et al. (2019) | Abcam | 28-8 | 1: 100 | NA | Tumor cells and immune cells | NA |
| Ono et al. (2018) | NA | NA | NA | Specific level > 0 | Tumor cells | NA |
| Roberts et al. (2017) | Cell Signaling Technology | E1L3N | 1: 200 | score ≥ 1 | Tumor cells and immune cells | NA |
| Rosery et al. (2021) | Dako | 22C3 | 1:40 | CPS ≥ 1 | Tumor cells and immune cells | CPS |
| Wang et al. (2019) | Abcam | 28-8 | 1: 50 | Specific score ≥ 3 | Tumor cells and immune cells | NA |
| Xing et al. (2020) | Dako | 22C3 | 1: 50 | TPS > 1% | Tumor cells | TPS |
| Yamashita et al. (2020) | Ventana/Roche | SP263 | Prediluted | CPS ≥ 1 | Tumor cells and immune cells | CPS |
| Yang et al. (2019) | Abcam | 28-8 | 1: 500 | Composite score ≥ 4 | Tumor cells | NA |
| Yao et al. (2021) | Dako | 28-8 | NA | > 1% | Tumor cells and immune cells | NA |

**Abbreviation:** PD-L1, programmed death ligand 1; TPS, tumor proportion score; CPS, combined positive score.

**sTable 4.** Subgroup analysis of selected pooled-results based on PD-L1 information.

| **Subgroup** | **Grade** | | | | **Histological differentiation** | | | | **PD-1 expression** | | | | **Overall survival** | | | |
| --- | --- | --- | --- | --- | --- | --- | --- | --- | --- | --- | --- | --- | --- | --- | --- | --- |
|  | Meta-analysis | | Heterogeneity | | Meta-analysis | | Heterogeneity | | Meta-analysis | | Heterogeneity | | Meta-analysis | | Heterogeneity | |
|  | OR (95% CI) | P-value | I² (%) | P-value | OR (95% CI) | P-value | I² (%) | P-value | OR (95% CI) | P-value | I² (%) | P-value | HR (95% CI) | P-value | I² (%) | P-value |
| **PD-L1 antibody clone** | 12.72 (0.23, 706.10) | 0.215 | 84 | 0.013 | 4.13 (1.24, 13.76) | 0.021 | NA | | 3.45 (1.40, 8.50) | 0.007 | 0 | 0.750 | 1.97 (0.68, 5.69) | 0.210 | 0 | 0.930 |
|  | 8.99 (1.61, 50.15) | 0.012 | 0 | 0.465 | 4.06 (1.03, 16.06) | 0.046 | NA | | NA | | | | 1.62 (1.12, 2.35) | 0.010 | 0 | 0.627 |
|  | 4.84 (1.43, 16.41) | 0.011 | 53.9 | 0.141 | 10.31 (3.10, 34.27) | 0.000 | NA | | NA | | | | NA | NA | NA | |
|  | 0.17 (0.02, 1.49) | 0.109 | NA | | 0.17 (0.02, 1.49) | 0.109 | NA | | 1.80 (0.15, 21.48) | 0.642 | NA | | NA | | | |
|  | 1.97 (1.03, 3.74) | 0.039 | 24.8 | 0.262 | 1.51 (0.68, 3.38) | 0.312 | 11 | 0.289 | 5.36 (1.45, 19.74) | 0.012 | NA | | 2.38 (1.59, 3.57) | 0.000 | 0 | 0.528 |
|  | 7.35 (4.21, 12.81) | 0.000 | NA | | NA | | | | 6.55 (1.19, 36.04) | 0.031 | NA | | 0.85 (0.28, 2.58) | 0.773 | 76.1 | 0.015 |
|  | 9.69 (2.73, 34.41) | 0.000 | NA | | 9.69 (2.73, 34.41) | 0.000 | NA | | NA | | | | 2.47 (0.57, 10.76) | 0.228 | NA | |
| **PD-L1 cutoff definition** | 1.87 (0.67, 5.21) | 0.231 | 51.5 | 0.067 | 0.91 (0.15, 5.40) | 0.919 | 69.4 | 0.038 | 2.96 (1.17, 7.50) | 0.022 | 0 | 0.669 | 1.71 (1.23, 2.38) | 0.001 | 0 | 0.883 |
|  | NA | | | | 4.13 (1.24, 13.76) | 0.021 | NA | | NA | | | | 1.61(0.93, 2.80) | 0.326 | NA | |
|  | NA | | | | NA | | | | 5.71 (1.53, 21.32) | 0.01 | 0 | 0.802 | 0.47 (0.24, 0.92) | 0.029 | 0 | 0.603 |
|  | 5.40 (2.53, 11.53) | 0.000 | 72.6 | 0.003 | 5.31 (1.56, 18.01) | 0.007 | 74.1 | 0.021 | 5.36 (1.45, 19.74) | 0.012 | NA | | 2.38 (1.59, 3.57) | 0.000 | 0 | 0.528 |
|  | 22.62 (1.01, 506.66) | 0.049 | NA | | NA | | | | NA | | | | 2.47 (0.57, 10.76) | 0.228 | NA | |
| **Tumor compartments for PD-L1 testing** | 2.60 (0.80-8.46) | 0.113 | 68.8 | 0.012 | 1.79 (0.30, 10.56) | 0.522 | 81.6 | 0.004 | 3.61 (1.70, 7.70) | 0.001 | 0 | 0.702 | 1.84 (1.37, 2.48) | 0.000 | 0 | 0.607 |
|  | 5.71 (0.69, 46.99) | 0.105 | NA | | 4.06 (1.03, 16.06) | 0.046 | NA | | NA | | | | 1.95 (0.85, 4.47) | 0.114 | NA | |
|  | 4.63 (2.06, 10.41) | 0.000 | 68.6 | 0.004 | 3.42 (0.83, 14.00) | 0.088 | 70.3 | 0.035 | 5.71 (1.53, 21.32) | 0.010 | 0 | 0.802 | 1.24 (0.51, 2.98) | 0.635 | 72.1 | 0.006 |
| **PD-L1 scoring system** | 2.31 (0.61, 8.76) | 0.220 | NA | | NA | | | | 3.21 (1.18, 8.75) | 0.023 | NA | | 2.10 (1.02, 4.31) | 0.043 | 0 | 0.87 |
|  | 3.63 (1.78, 7.42) | 0.000 | 69.2 | 0.000 | 2.22 (0.90, 5.51) | 0.084 | 65.0 | 0.014 | 4.33 (1.58, 11.87) | 0.004 | 0 | 0.744 | 1.95 (1.50, 2.54) | 0.000 | 0 | 0.742 |
|  | 9.10 (2.76, 29.95) | 0.000 | NA | | 10.31 (3.10, 34.27) | 0.000 | NA | | NA | | | | NA | | | |
|  | NA | | | | NA | | | | 6.55 (1.19, 36.04) | 0.031 | NA | | 0.47 (0.24, 0.92) | 0.029 | 0 | 0.603 |

**Abbreviation:** NA, not available; TPS, tumor proportion score; CPS, combined positive score; PD-1, programmed cell death protein 1.

**sFigure 1.** Funnel plot showing the correlation between PD-L1 expression and various indicators in patients with gastroenteropancreatic neuroendocrine neoplasms. (A) Gender, (B) Age, (C) Grade, (D) Histological differentiation, (E) Pathology, (F) Stage, (G) Invasion, (H) Metastasis, (I) PD-1 expression, (J) CD8 expression, (K) FOXP3 expression, (L) MMR status, (M) OS.


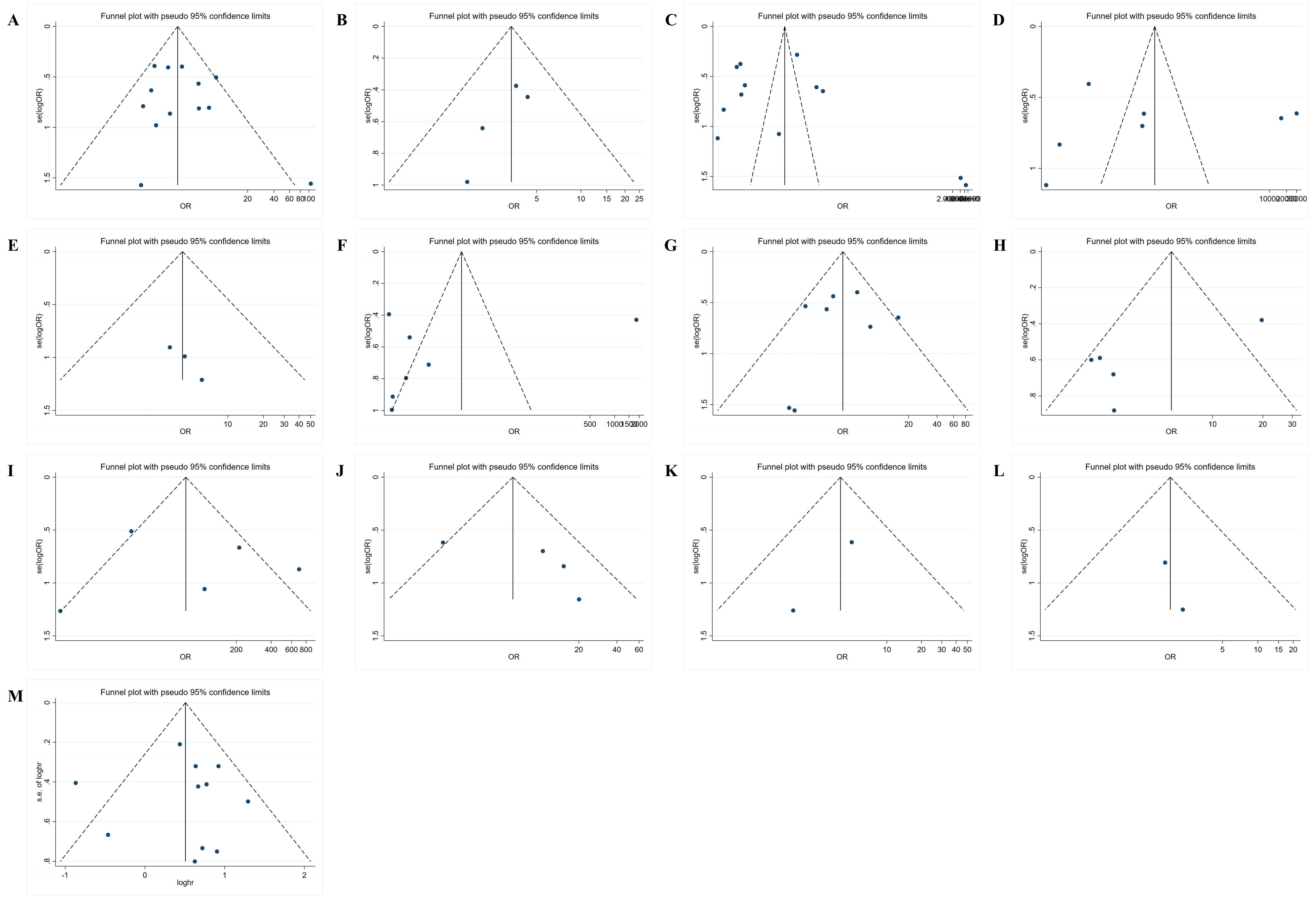


**sFigure 2.** Sensitivity analysis showing the correlation between PD-L1 expression and various indicators in patients with gastroenteropancreatic neuroendocrine neoplasms. (A) Gender, (B) Age, (C) Grade, (D) Histological differentiation, (E) Pathology, (F) Stage, (G) Invasion, (H) Metastasis, (I) PD-1 expression, (J) CD8 expression, (K) FOXP3 expression, (L) MMR status, (M) OS.


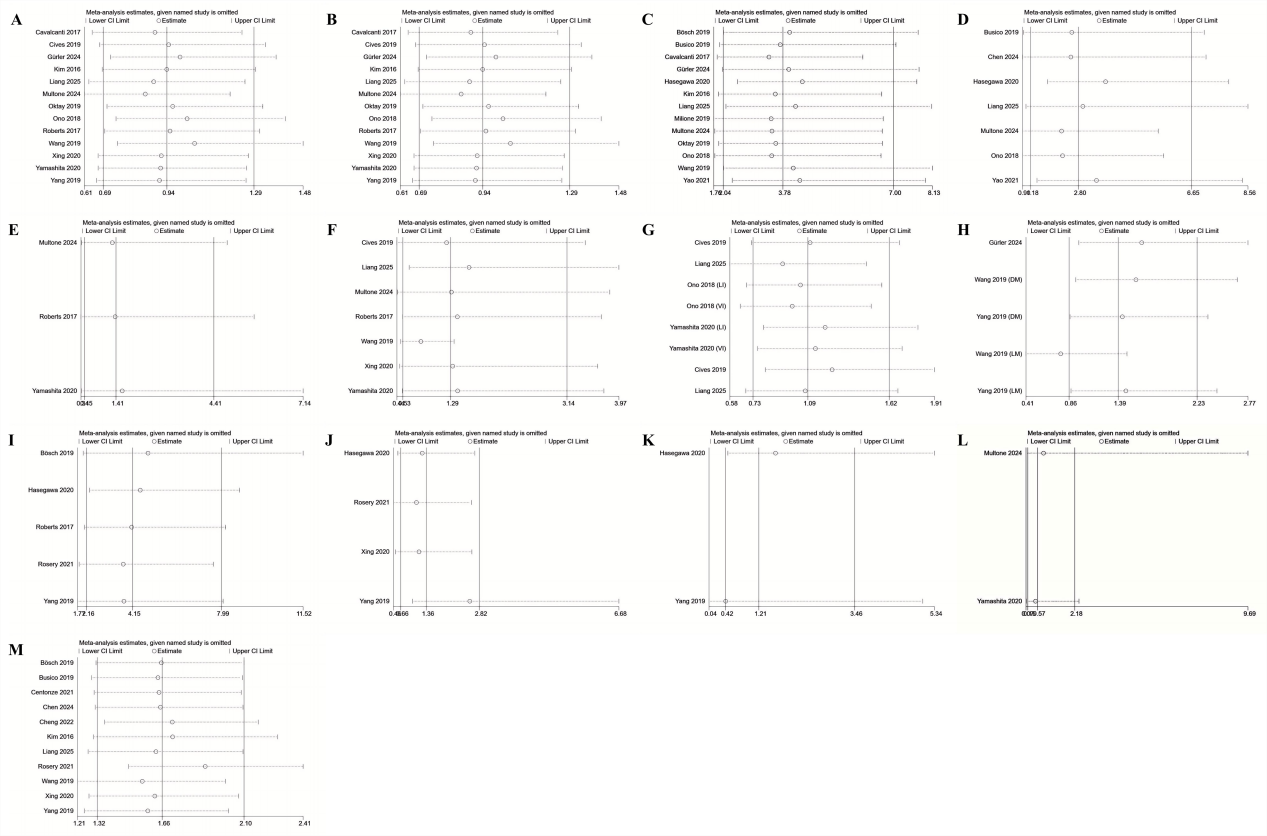


**Abbreviation:** DM, distant metastasis; LI, lymphatic invasion; LM, lymphatic metastasis ; VI, venous invasion.
